# In Vitro Fertilization (IVF) Outcomes in Patients With Endometriosis Compared to Patients With Tubal Factor Infertility in Indonesia: A Retrospective Study

**DOI:** 10.7759/cureus.70668

**Published:** 2024-10-01

**Authors:** Mifta Nurindah, Hariyono Winarto, Mila Maidarti, R Muharam, Achmad Kemal Harzif, Budi Wiweko, Atika Mahira Yanfaunnas, Gita Pratama

**Affiliations:** 1 Department of Obstetrics and Gynecology, Faculty of Medicine, Universitas Indonesia, Jakarta, IDN; 2 Gynecology Oncology Division, Department of Obstetrics and Gynecology, Faculty of Medicine, Universitas Indonesia, Jakarta, IDN; 3 Reproductive Immunoendocrinology Division, Department of Obstetrics and Gynecology, Faculty of Medicine, Universitas Indonesia, Jakarta, IDN; 4 Yasmin In Vitro Fertilization (IVF) Clinic, Dr. Cipto Mangunkusumo General Hospital, Jakarta, IDN; 5 Human Reproduction, Infertility, and Family Planning Cluster, Indonesia Reproductive Medicine Research and Training Center, Faculty of Medicine, Universitas Indonesia, Jakarta, IDN

**Keywords:** assisted reproductive technology, endometriosis, infertility, ivf, pregnancy rate

## Abstract

Background: Endometriosis is a disease characterized by dysmenorrhea, chronic pelvic pain, and infertility. The pathogenesis of endometriosis and its relationship to infertility are still not fully understood. In vitro fertilization (IVF) is considered an effective treatment for patients with endometriosis-associated infertility. This study compared the pregnancy rates of endometriosis patients who underwent IVF with those of patients with tubal obstruction.

Methods: This was a retrospective cross-sectional study of 225 patients with endometriosis and tubal factor infertility who underwent IVF at Yasmin Clinic, Dr. Cipto Mangunkusumo National General Hospital, Jakarta, Indonesia, between January 2013 and August 2021. Demographic data, anti-Mullerian hormone (AMH) levels, the initial and total dose of recombinant follicle stimulating hormone (rFSH), the total dose of recombinant luteinizing hormone (rLH), stimulation duration, oocyte maturation rates, fertilization rates, embryo cleavage rates, and pregnancy rates (biochemical, clinical and ongoing) were obtained.

Results: AMH level, number of oocytes retrieved, and embryo cleavage rates were significantly lower in the endometriosis group. Initial and total doses of rFSH and total dose of rLH were higher, and the duration of stimulation was longer in the endometriosis group compared to the tubal factor group. In the endometriosis group, the biochemical (47.3% vs. 52.7%, p=0.375), clinical (43.1% vs. 56.9%, p=0.215), and ongoing (45.5% vs. 54.5%, p=0.511) pregnancy rates were lower than those in the tubal factor group. However, there were no statistically significant differences between the two groups. There was no significant difference in pregnancy rates between the short antagonist and ultra-long protocols (three months of downregulation). From multivariate analysis, only rLH supplementation was found to be significantly related to pregnancy outcomes in patients with endometriosis.

Conclusion: AMH levels, number of oocytes retrieved, and embryo cleavage rates were lower in patients with endometriosis. However, pregnancy rates were not significantly different from patients with tubal factors. Supplementation with rLH may improve pregnancy rates in patients with endometriosis who have undergone IVF programs.

## Introduction

Endometriosis is a disease characterized by the presence of endometrial-like tissue outside the uterine cavity and clinically associated with many symptoms such as dysmenorrhea, chronic pelvic pain, and infertility [1]. The disease affects approximately 10% of young women, representing approximately 200 million women of childbearing age, and increases to 50% in patients with chronic pelvic pain, infertility, or both [2,3]. In Indonesia, the prevalence rate of endometriosis has yet to be estimated. Endometriosis causes a significant economic burden and indirect costs, such as loss of work productivity [4].

The pathogenesis of endometriosis and its relationship to infertility is still unclear. Several studies have revealed that endometriosis can interfere with fertility through mechanical and biochemical mechanisms. First, a pelvic adhesion mechanically causes anatomical (tubal-ovarian) distortion, thereby interfering with the release of oocytes from the ovaries, oocyte retrieval, and oocyte transport by the fallopian tubes. Second, endometriosis biochemically changes the peritoneal environment to be rich in inflammatory cytokines, resulting in poor-quality oocytes, changes in endometrial receptivity, and implantation failure [2,5].

According to the European Society of Human Reproduction and Embryology (ESHRE) guidelines, in vitro fertilization (IVF) is an effective treatment and is often offered to women with endometriosis-associated infertility [6]. IVF is also a safe procedure with few complications in patients with endometriosis. Studies have shown several factors that influence the success rate of IVF in patients with endometriosis, including the patient’s age, severity of endometriosis, anti-Mullerian hormone (AMH) level, oocyte and embryo quality, endometrial receptivity, and history of previous surgery [7,8]. The effect of endometriosis on the success of IVF is still debated. Some studies showed lower pregnancy rates of women with endometriosis while other studies found no difference between endometriosis and other causes of infertility [5].

Currently, there is no data about IVF outcomes of endometriosis patients in Indonesia. Therefore, this study aimed to compare the pregnancy rate among patients with endometriosis and tubal factor infertility who had undergone IVF at the Yasmin Clinic, Dr. Cipto Mangunkusumo National General Hospital, Jakarta, Indonesia.

## Materials and methods

The Research Ethics Committee of the Faculty of Medicine, University of Indonesia, approved the study (KET-721/UN.F1/ETIK/PPM.00.02/2019). The researcher guarantees the confidentiality of all patient information taken from the patient's medical records, and the data obtained was only used for research purposes and displayed at the time of the presentation of the study results.

A case-control study was performed on women who underwent IVF with a total number of 225 subjects. Patient data were taken from medical records. The inclusion criteria for this research were women aged under 38 years who were diagnosed with endometriosis through anamnesis, physical examination, and transvaginal ultrasound. Subjects with infertility due to male factors, polycystic ovarian syndrome (PCOS), uterine malformations, or immune conditions were excluded from the study. The control group included infertile women with tubal problems who underwent IVF.

Clinical data from the subjects included age, body mass index, AMH level, duration and type of infertility. The initial dose of recombinant follicle stimulating hormone (rFSH), total dose of rFSH, total dose of recombinant luteinizing hormone (rLH), duration of stimulation, number of oocytes, oocyte maturation rates, fertilization rates, embryo cleavage rates, and pregnancy rates (biochemical, clinical, and ongoing) were analyzed.

After the data were collected, data verification, editing, and coding were conducted. Data analysis was performed using the SPSS version 26.0 computer statistical program (IBM Corp., Armonk, New York, USA). Data were presented in tabular form along with a narrative. Data were presented in the form of mean ± standard deviation if the data distribution is normal, but if the data distribution is not normal, the data is presented in the form of median (minimum-maximum range). Eligible variables will be used as variables in multivariate data analysis using multinomial logistic regression.

All patients underwent freeze embryo transfer (FET), an assisted reproductive technology where previously cryopreserved embryos are thawed and transferred into the uterus. Before the transfer, hormonal treatments were administered, as mentioned earlier. The process includes preparation of the uterine lining, thawing of embryos, embryo selection, and the actual transfer guided by ultrasound. 

Additional analysis was conducted on the controlled ovarian stimulation methods used in the endometriosis group. Out of 112 endometriosis patients, 100 were given the short protocol, while 12 received the long protocol. The endometriosis patients who underwent IVF were also analyzed to the variables and pregnancy outcomes.

## Results

Two hundred twenty-five subjects were included (112 endometriosis and 113 tubal factor infertility) in this study. The baseline characteristics of the subjects can be found in Table 1. Normally distributed data are presented as mean ± standard deviation, otherwise presented as median. Age, BMI, length of infertility, and type of infertility did not significantly differ between the two groups. Nonetheless, a statistically significant difference (p=0.004) in AMH levels was observed between the two groups.

**Table 1:** Characteristics of women with endometriosis and tubal factor infertility. *p<0.05. BMI: body mass index; AMH: anti-Mullerian hormone. a: presented as mean (±SD); b: presented as N (%); c: presented as median (range).

Characteristics	Endometriosis (n=112)	Tubal factor infertility (n=113)	p-value
Age (years)^a^	36.55 ± 5.7	37.95 ± 5.4	0.583
<31^b^	36	39	
31-35^b^	49	42	
36-40^b^	27	32	
>40^b^	0	0	
BMI (kg/m^2^)^c^	23.3 (17.3–37.6)	23.1 (18–38.6)	0.812
AMH (ng/mL)^a^	2.1 ± 1.5	3.4 ± 3.1	0.004*
Infertility duration (years)^c^	6 (1–18)	5 (1–18)	0.443
Infertility type			
Primary^b^	106 (94.6%)	100 (88.5%)	0.181
Secondary^b^	6 (5.4%)	13 (11.5%)	

In women with endometriosis, both the initial and total rFSH doses and rLH doses for ovarian stimulation were higher than those in patients with tubal infertility. However, the number of retrieved oocytes and embryo cleavage rates were significantly lower in patients with endometriosis (Table 2). No statistically significant differences were found in oocyte maturation or fertilization rates between the two groups. In the endometriosis group, biochemical, clinical, and ongoing pregnancy rates were lower than those in the tubal infertility group. However, the two groups were not significantly different.

**Table 2:** IVF outcomes of women with endometriosis and tubal factor infertility. *p<0.05 BMI: body mass index; AMH: anti-Mullerian hormone; rFSH: recombinant follicle stimulating hormone; rLH: recombinant luteinizing hormone; IVF: in vitro fertilization. a: presented as mean (±SD); b: presented as N (%); c: presented as median (range).

Outcome	Endometriosis (n=112)	Tubal factor infertility (n=113)	p-value
Initial dose rFSH (IU)^a^	283.8 ± 79.3	255.1 ± 67.1	0.013*
Total dose rFSH (IU)^a^	3270.5 ± 1419.2	2768.2 ± 1347.3	0.017*
Total rLH dose (IU)^a^	622.9 ± 755.7	423.8 ± 312.8	0.036*
Stimulation duration (days)^c^	11 (7–17)	11 (8–22)	0.091
Number of oocytes^ a^	8 ± 6	11 ± 7	0.000*
Oocyte maturation rate (%)^c^	92.8 (30–100)	87.6 (46–100)	0.815
Fertilization rate (%)^c^	66.7 (16.7–100)	68.8 (16.7–100)	0.966
Embryo cleavage rate (%)^c^	97.3 ± 10.4	99.4 ± 5.1	0.046*
Biochemical pregnancy^b^	72 (64.3%)	77 (68.1%)	0.375
Clinical pregnancy^b^	35 (31.2%)	41 (36.3%)	0.215
Ongoing pregnancy^b^	22 (19.6%)	24 (21.2%)	0.511

We also analyzed controlled ovarian stimulation methods used in the endometriosis group; all were given single stimulation. The majority of the patients in the endometriosis group (100 (89.3%)) were given a short protocol, while the rest used an ultra-long protocol. In short protocols, GnRH antagonists’ daily injection for suppressing LH was started when the growing follicles were 12 mm. While in the ultra-long protocol, long-acting GnRH agonists were injected approximately three months before rFSH administration. However, there was no statistical difference between the short and long protocol in terms of pregnancy outcomes in the endometriosis group (Table 3).

**Table 3:** Pregnancy outcomes by type of stimulation in endometriosis patients. *p<0.05. a: presented as N (%).

Pregnancy outcome	Short protocol (N=100)	Long protocol (N=12)	P-value
Biochemical pregnancy^a^	64 (64.0%)	8 (66.7%)	1.000
Clinical pregnancy^a^	31 (31.0%)	4 (33.3%)	1.000
Ongoing pregnancy^a^	22 (22%)	2 (16.7%)	0.687

As an additional analysis, an assessment of the relationships between variables and pregnancy outcomes in the endometriosis group undergoing IVF was carried out (Table 4). There was no significant relationship between the variables in the endometriosis subject group and clinical pregnancy outcomes in IVF. As an additional analysis, an assessment of the relationships between variables and pregnancy outcomes in the endometriosis group undergoing IVF was carried out (Table 4). There was no significant relationship between the variables in the endometriosis subject group and clinical pregnancy outcomes in IVF.

**Table 4:** Bivariate analysis of the relationships between endometriosis subject characteristics and pregnancy outcomes in IVF. **Fisher’s exact test. BMI: body mass index; AMH: anti-Mullerian hormone; rFSH: recombinant follicle stimulating hormone; rLH: recombinant luteinizing hormone; IVF: in vitro fertilization. a: presented as mean (±SD); b: presented as N (%); c: presented as median (range).

Outcome	Endometriosis	P-value
Age (years)^c^	32 (26–38)	0.522
BMI (kg/m^2^)^a^	23.79 (17.3–37.6)	0.337
AMH (ng/mL)^a^	2.1 ± 1.6	0.492
Duration of infertility (years)^c^	6 (1–18)	0.178
Initial dose of rFSH (IU)^a^	289.58 ± 76.368	0.106
Total dose of rFSH (IU)^a^	3756.94 ± 1137.692	0.084
Total dose rLH (IU)^a^	585 ± 810.710	0.154
Stimulation duration (days)^c^	11 (7–17)	0.380
Number of oocytes picked^a^	9 ± 6	0.981
Oocyte maturation rate (%)^c^	89.76 (50–100)	0.818
Fertilization rate (%)^c^	62.55 (16.6–100)	0.638
Embryonic cleavage rate (%)	93.87 ± 18.08	0.808
Stimulation type**		0.522
Infertility type**		0.254

The variables that were eligible for multivariate analysis (p<0.25) were the duration of infertility, type of stimulation, total dose of rFSH, and total dose of rLH (Table 5). According to the results of the multivariate analysis, the rLH dose was significantly related to the clinical pregnancy outcome during IVF in the endometriosis group (p=0.015).

**Table 5:** Relationships between endometriosis subject characteristics and pregnancy outcomes in IVF patients according to multivariate analysis. *p<0.05. BMI: body mass index; AMH: anti-Mullerian hormone; rFSH: recombinant follicle stimulating hormone; rLH: recombinant luteinizing hormone, IVF: in vitro fertilization.

Variable	Coefficient beta	P-value	Odds ratio	95% CI
Duration of infertility (years)	0.017	0.826	1.017	0.87–1.184
Stimulation type	0.002	0.563	1.002	0.994–1.010
Total dose of rFSH	0.000	0.67	1.0	1.000–1.001
rLH dose	–0.002	0.015*	0.998	0.996–1.000

## Discussion

In this study, we found that endometriosis patients required higher doses of rFSH during stimulation compared to the control group. This result is in accordance with a previous study, which showed a tendency toward more days and higher dosages of rFSH in endometriosis patients than in tubal factor infertility patients [2]. Stimulation with rFSH has been used as a companion to IVF to stimulate the ovaries for increased success. However, increased rFSH dose is also related to a negative impact on the oocyte quality and endometrial receptivity, as well as an increased risk of ovarian hyperstimulation syndrome (OHSS) [9]. Gonzalez-Fernandez et al. demonstrated that expression of the FSH receptor (FSHR) was positively correlated with Cyp 19A1 and pregnancy-associated plasma protein-A (PAPP) in infertile women without endometriosis. However, PAPP was not correlated with FSHR in endometriosis women, like what has been found in poor responder patients. This phenomenon can cause disruption of the FSH receptor signaling pathway, hence requiring higher doses of rFSH to stimulate follicle development [10].

There was no significant difference in the number of mature oocytes between the two groups. The results of this study are consistent with those of various other studies. Age-independent factors, such as type of infertility, did not necessarily affect oocyte quality. Juneau et al. supported this association by comparing abnormal oocyte ratios in age-adjusted endometriotic and non-endometriotic groups [11]. Furthermore, research conducted by Robin et al. revealed no discernible differences in the quantity of mature oocytes or their morphology [12]. A cohort study revealed fewer mature oocytes with normal morphology in the endometriosis group. However, the debate regarding the influence of oocyte morphology on IVF outcomes persists [13].

A study by Ashrafi et al. revealed that oocyte dysmorphism may influence fertilization rates, yet it does not seem to have a significant effect on implantation or pregnancy rates during IVF [14]. A meta-analysis showed that the absence of four oocyte structural abnormalities-first polar body, increased perivitelline space, refractive body, and intracytoplasmic vacuole-was substantially correlated with the fertilization rate [15]. Additional research suggests that the developmental potential of embryos may be affected by the thickness of the zona pellucida. A literature review revealed that the zona pellucida and perivitelline space significantly affect oocytes. A larger perivitelline space correlates with heightened probabilities of acquiring high-quality embryos [16].

In our investigation, we observed a reduced embryo cleavage rate among individuals with endometriosis, which corresponds to the results reported by Huang et al. Their study similarly demonstrated that endometriosis patients exhibited a decreased proportion of high-quality cleavage-stage embryos compared to the control group [17]. In contrast, a study by Sanchez et al. revealed no difference in the embryo cleavage rate between women with and without endometriosis [18]. The difference in pregnancy success due to fertilization rate was not significant. A literature search corroborates this result, with various studies showing similar results. A systematic review and meta-analysis showed that endometriosis significantly reduces the fertilization rate in IVF. Endometriosis had a negative effect on the number of mature oocytes in the IVF cycle compared to that in the control group; this is caused by an increase in reactive oxidative species (ROS), which can affect follicle maturation, resulting in oocyte DNA damage [19].

This study revealed statistically significant differences in AMH levels between the endometriosis and tubal factor infertility groups. However, further investigations revealed that low AMH levels, even in severe endometriosis, did not affect oocyte quality or pregnancy outcome despite reducing oocyte numbers [20]. Hsieh et al. also reported that AMH levels were age dependent. In this study, the effect of decreasing AMH levels with age was 39 years. One of the exclusion criteria for this study was age >38 years. The mean and median ages of the research subjects were 33.2 and 35 years, respectively [21]. A study by Maidarti et al., which revealed a significant association between AMH levels and pregnancy success, also used a population similar to that of this study [22].

Descriptive analysis with cross-tabulation showed that the pregnancy rate in the endometriosis group was lower than that in the tubal factor infertility group, with a greater proportion of biochemical pregnancies than clinical or current pregnancies. However, there was no statistically significant difference between the pregnancy proportions in each group. These results are consistent with the pathophysiology of endometriosis, namely, pituitary-ovarian axis dysfunction, impaired folliculogenesis, inhibition of ovulation, decreased luteal cell function, decreased oocyte cell quality, embryo abnormalities, implantation failure, increased oxidative stress and inflammation that damage the embryo and sperm and decreased expression of endometrial receptivity factors [1,19]. Further literature review also showed that the concordance of these results could be found in other studies.

A cohort study by Nirgianakis et al. revealed a significantly increased risk and complication of pregnancy in the endometriosis group [23]. There was no significant association between endometriosis and tubal factor infertility in the Glavind et al. cohort study, which had a larger sample size [24]. One meta-analysis also revealed an association between the two factors, but with weaker strength [25]. Another meta-analysis also revealed significant associations, such as pre-eclampsia, gestational hypertension, placental abruption, stillbirth, preterm birth, and placental previa [26]. The Society for Assisted Reproductive Technology (SART) database comparing successful pregnancy in the endometriosis and tubal factor infertility groups had the same results as this study, namely, a nonsignificant difference between the endometriosis and tubal factor infertility groups in terms of the success of IVF pregnancy [27].

Multivariate analysis revealed that supplementation with rLH during stimulation was the only factor influencing pregnancy rates in the endometriosis group. As mentioned above, the total rFSH was higher in the endometriosis group. It indicates that women with endometriosis have a poorer response to stimulation than women with tubal infertility. Supplementation with rLH has long been carried out to improve ovarian response during stimulation, especially in poor responder groups and women aged over 35 years. LH is important for steroidogenesis and follicle development, especially androgen production in theca cells, conversion into estrogen in granulosa cells and achieving oocyte competence prior to oocyte retrieval. However, its levels are sometimes very low during ovarian stimulation due to GnRH suppression by both agonists and antagonists. Several studies have shown that rLH administration is associated with increased oocyte, embryo quality and pregnancy rates in poor responder and advanced-age women [28,29]. However, the effect of rLH administration in patients with endometriosis who have undergone IVF is not yet known.

A meta-analysis conducted by Liu et al. showed that downregulation for three months increased the clinical pregnancy rate in women with endometriosis with an RR of 1.31 when compared with the long protocol. Long-term administration of GnRH agonists is thought to reduce inflammation caused by endometriosis, thereby improving oocyte quality, and restoring endometrial receptivity [30]. However, our study found no significant differences in biochemical, clinical, and ongoing pregnancies between ultra-long and short protocols used in the endometriosis group. This may be due to the small number of patients with the ultra-long protocol in this study, so subgroup analysis could not be carried out properly. The use of the ultra-long protocol in women with endometriosis is a promising method and requires further research, especially in various degrees of severity of endometriosis and also adenomyosis.

This study has several limitations. Using secondary data in the form of medical records presents challenges in observing the epidemiology of endometriosis cases. Due to the study's retrospective nature, some missing data could not be retrieved and were excluded from the analysis. Consequently, there were relatively few subjects in this study, which was conducted at only one hospital, so the findings may not be generalizable to a larger population. Additionally, the study focuses on two pathologies; it is recommended to include another group for comparison.

## Conclusions

In conclusion, several characteristics were found to be lower in patients with endometriosis, including AMH levels, the number of oocytes, and embryo cleavage rates compared with tubal factor infertility. Additionally, women with endometriosis exhibited lower biochemical, clinical, and ongoing pregnancy rates, although they were not statistically significant. However, it seems that supplementation with rLH significantly improved pregnancy rates in the endometriosis group. To further elaborate, these findings suggest that while endometriosis patients may experience lower pregnancy rates overall, the influence of recombinant luteinizing hormone (rLH) could play a crucial role in improving their chances of conception. This finding underscores the importance of considering hormonal factors in the management and treatment of infertility associated with endometriosis.
